# Utilizing Competency-Based Education to Evaluate the Research Skills of Nursing Students: A Systematic Review and Meta-Analysis

**DOI:** 10.7759/cureus.62549

**Published:** 2024-06-17

**Authors:** Norah G Alkhaledi, Sarah A Alabdalhai, Nasima Y Awaji, Omar G Baker, Ali M Alyasin, Bushra J Al Hnaidi, Abdulrahman S Alayed, Yasir O Ashour

**Affiliations:** 1 Nursing, Umm Al-Qura University, Makkah, SAU; 2 Nursing, King Saud University, Riyadh, SAU

**Keywords:** nursing skills, basic research, research skills, nursing students, competency-based education

## Abstract

Exploring and implementing competency-based education approaches to assess research skills are necessary to close the gap between research and practice, promote lifelong learning among future nurses, and improve research literacy. This study aims to assess the effectiveness of competency-based education in improving the assessment of research skills among nursing students. A systematic review and meta-analysis of the literature was performed following the Preferred Reporting Items for Systematic Reviews and Meta-Analyses (PRISMA) guidelines.

Population, Intervention, Comparison, Outcomes and Study (PICOS) eligibility criteria were used to select original studies published between 2017 and 2023. As a first step in the data-handling process, titles and abstracts of all articles retrieved by the search strategy were screened for relevance, and the irrelevant articles were discarded. The screening process was conducted by two authors independently, and the final decisions were made together. A meta-analysis was performed to assess the effectiveness of competency-based education in improving the assessment of research skills among nursing students. Five quantitative studies were appraised using the Joanna Briggs Institute checklist. The effect size was 0.69 ± 0.35 (P = 0.05), which indicates a high effect on research competency among nursing students who attend courses or training in research matters, after transforming data to correlation coefficient resulting in r = 0.5.

The study encourages research literacy among nursing students. Through competency-based learning, students are exposed to a variety of research methodologies, ethical issues, and scientific writing conventions. This exposure enhances their capacity to understand, assess, and apply research evidence, empowering them to become knowledgeable consumers and field contributors. While making evidence-based decisions, nurses with research competencies can actively incorporate the most recent research findings into their clinical practice. Furthermore, nurses with strong research abilities can influence health policy and practice.

## Introduction and background

Nurses are fundamental to the healthcare system and essential to patient care and health promotion. Therefore, nursing research is crucial in advancing nursing knowledge, enhancing patient outcomes, and influencing practice. As nursing has expanded outside of the hospital to include communities, businesses, home care, and schools, the position of a nurse has undergone a significant change with the goal of delivering superior, evidence-based practice care [1]. Research provides the scientific foundation for nursing practice. Nursing research focuses on understanding and easing the symptoms of acute and chronic illness, identifying practical ways to achieve and maintain optimal health, improving the clinical settings in which care is provided, and analyzing phenomena related to nursing, including healthcare delivery, nursing education, administration, and nurse traits and roles [1].

In Saudi Arabia, the Saudi Vision 2030 plan aims to bring higher education up to par with other global standards by prioritizing research and development because Saudi Arabia is among the top 50 countries in the world with the most significant proportion of scientific research [1]. Nursing, however, contributed less research than other fields in Saudi Arabian health sciences research, accounting for 1.6% of all publications [1,2].

In the field of nursing, evidence-based practice (EBP) is a crucial element and advantageous result of research studies, which necessitates a deep understanding of the research methodology [3]. Consequently, EBP allows nurses to examine research to gain a deeper understanding of the benefits and outcomes of therapeutic assessments and interventions. Research is a course that takes a comprehensive approach to education, both in terms of how it is delivered and how students ought to be assessed in accordance with the learning objectives [2,3]. Courses that require advanced and complex methods, like research, should be taught using the competency-based education (CBE) framework to meet the necessary learning objectives [3]. Conceptually, CBE is a relatively new educational paradigm that has been applied in several educational settings. It has been embraced by numerous universities and academic fields and, as a result, has grown to be a major force for innovation and transformation in the fields of medicine and higher education [3].

To our knowledge, this is a rare study in Saudi Arabia as a systematic review and meta-analysis in utilizing CBE to evaluate nursing students' research skills. Research-based learning methods for the evaluation of research skills that are competency-based need to be investigated and implemented to enhance research literacy, reduce the gap between research and practice, and promote lifelong learning among future nurses.

Problem statement

The study addresses the pressing need for improving research literacy and bridging the gap between research and practice in nursing education. By exploring and implementing CBE approaches for assessing research skills, this study seeks to enhance the competence levels of nursing graduates and empower them to effectively interact with research evidence, ultimately contributing to evidence-based care. Through a systematic review and meta-analysis, the study aims to provide valuable insights and recommendations for implementing comprehensive and uniform methods to evaluate nursing students' research skills. By doing so, this research endeavors to promote lifelong learning among future nurses and ensure that they possess the necessary research skills to meet the demands of evidence-based practice [4].

Significance of the study

Nursing education and research will be greatly impacted by the study on competency-based learning's assessment of nursing students' research skills. It provides an organized structure for assessing nursing students' research skills, to start. With the integration of particular research-related competencies, competency-based learning guarantees that students are skilled in areas like literature review, study design, data collection, analysis, and dissemination [5].

Additionally, the study encourages nursing students to be research literate. Students are exposed to a range of research methodologies, ethical considerations, and scientific writing conventions through competency-based learning. Their ability to comprehend, evaluate, and apply research evidence is improved by this exposure, enabling them to become informed consumers and field contributors [6].

Research question

Among nursing students, does the implementation of CBE lead to improved assessment of research skills?

Background

Over the past few years, competency-based programs have rapidly expanded throughout higher education, and this trend is expected to continue [7]. Although the concept of CBE dates back a long way, it has only been put into practice recently in several educational settings. It is being embraced by numerous disciplines and institutions of higher learning, but it has emerged as a significant driver of innovation and transformation in medical education [8]. A student must demonstrate that they have achieved a set of learning outcomes, which is known as CBE. Terms like competency-based, mastery-based, outcome-based, performance-based, and standards-based education are interchangeable with CBE [9].

Nursing bachelor's degree holders who work full-time are typically the target audience for competency-based training programs in nursing. CBE limits the learning options of students by stressing practical training, which discourages true learning even as it promotes learning that is significant to the market [3-9]. Although it has been gradual, the acceptance of CBE, which is not based on credit hours, in nursing education is growing. Nonetheless, there appears to be a lack of recognition for CBE in nursing education, indicating notable deficiencies in the body of research in this field. Positively, all parties involved need to collaborate to create a standard method for competency evaluation in nursing education [9].

Around the world, nursing education has changed to a competency-based curriculum as part of the shift from "training" to "education." Therefore, the emphasis on student performance and learning outcomes to accomplish desired results and pedagogical standards is the hallmark of CBE [3-9]. As CBE models grow, it will be more crucial for nurses to assess competency, identify the health requirements of the populations they serve, decide capabilities, and create adaptable, self-regulated learning strategies [9].

CBE is demanding and offers an educational paradigm that promotes learning [9]. The students now view CBE as a useful educational approach that would enable them to fulfill their dream of earning a college degree, which may have previously been out of reach for them [3-9]. As each educational program is customized to the needs of the student and includes interventions aimed at meeting their unique needs, the author lauded the efficacy of CBE in assessing the research skills of nursing students. In the CBE program, students often work at their own pace and have unique access to webinars, interactive activities, videos, simulations, and practice exams related to any courses assigned [3-9].

The study aims to assess the effectiveness of CBE in improving the assessment of research skills among nursing students through systematic review and meta-analysis.

## Review

Methods

Research Design

A systematic review and meta-analysis of the literature was performed following the Preferred Reporting Items for Systematic Reviews and Meta-Analyses (PRISMA) guidelines.

When performing a meta-analysis study, inclusion and exclusion criteria are essential elements. These criteria help researchers in defining the characteristics of studies that will be taken into consideration for inclusion or exclusion from the analysis (Table 1).

**Table 1:** Inclusion and exclusion criteria

Inclusion criteria	Exclusion criteria
Quantitative studies and cross-sectional design	Studies that were not published in English
Studies that assess the research skills among nursing students	Commentaries, discussion papers, dissertations, narratives, opinion pieces, editorials, secondary analyses of preexisting data, qualitative studies, and studies that did not report on the assessment of research skills in nursing students through competency-based learning
Studies that were conducted among nursing students	
English language papers, published between 2017 and 2023	
Studies utilizing the Research Competency Scale for Nursing students (RCS-N) involved in the meta-analysis	

Search Strategy

The search was conducted in March 2024. The literature search to identify key search terms was developed by the researchers. The searches used the following Boolean operators and Medical Subject Headings (MeSH) search terms: “Nursing” OR “nursing student” OR “Nurse.”) AND (“research competencies” OR “research skills” OR “Research”) AND (“competency-based learning” OR “CBL”).

Three databases, namely ProQuest, PubMed, and Web of Science, were searched to find relevant studies. In a systematic review, searching more than two databases can help to increase the coverage of relevant studies, minimize bias, increase accuracy, and improve the process's quality and robustness [10]. The search results are limited to articles in English language and full-text availability.

Screening of Articles

As a first step in the data-handling process, titles and abstracts of all studies retrieved by the search strategy were screened for relevance, and all those that were irrelevant were discarded. As a second step, two review team members independently assessed the eligibility of the studies using the predefined inclusion and exclusion criteria. Any disagreements on whether to include a specific study were resolved by discussion between the reviewers. The third and final step in the data-handling process is quality control of the included studies using a quality assessment tool for quantitative research, namely the Joanna Briggs Institute (JBI) checklist [11].

Data Extraction

The identified records were exported to an MS Excel spreadsheet that was used for extraction followed by full-text screening. The following data were considered for extraction: (a) authors, (b) year of publication, (c) country of the study, (d) sample size, (e) study design, (f) main results, (g) intervention details, (h) outcome measures, and (i) limitations (Table 2).

**Table 2:** A summary of the included reviews

Authors/years	Study design	Participant count	Intervention	Main findings	Outcome measured	Intervention effects	Limitations
Toraman et al. [12], Turkey	Quantitative descriptive-comparative study	Total: 860	The methodology involved a quantitative descriptive-comparative study conducted in a university nursing department in Turkey, with 860 students enrolled and a 60% participation rate. Data collection was done through a paper-and-pencil technique in the classroom, following ethical approval.	Students taking a research course demonstrated significantly higher engagement in research activities, indicating a positive impact of the course on their participation in research-related tasks.	Participation in scientific meetings, involvement in research activities, participation in research as a subject, and collecting data on behalf of a researcher	The intervention effects of taking a nursing research course in the study showed significant positive effects on participation in scientific meetings, involvement in research activities, participation in research as a subject, and collecting data on behalf of a researcher. Students taking the research course demonstrated higher rates in all these aspects compared to students not taking the course.	The study was conducted in a specific region in Turkey, which may limit the generalizability of the findings to other regions or countries. The sample size of the study may not be large enough to capture all potential differences between nursing students taking a research course and those not taking the course. The study relied on self-report questionnaires, which could introduce bias or inaccuracies in responses. The study did not explore the long-term effects of taking a nursing research course on students' research involvement and attitudes. The study did not address potential confounding variables that could influence the outcomes observed. The study did not investigate the specific content or delivery methods of the nursing research course, which could impact its effectiveness.
Duru et al. [13], Turkey	The study design was a methodological study involving nursing personnel, including academic members and undergraduate students, using the Anxiety Scale Toward Research and the Attitude Scale Toward Scientific Research for criterion validity.	Total: 937 (Academic members: 422; undergraduate students: 515)	The intervention involved a methodological study of 937 nursing personnel, including academic members and undergraduate students. The Anxiety Scale Toward Research and the Attitude Scale Toward Scientific Research were used for criterion validity. The study was conducted in 10 steps, including various analyses and validations.	The study developed a valid and reliable Scientific Research Competency Scale with 57 items across four subdimensions, enabling the assessment of research competencies in nursing professionals at different education levels.	Scientific Research Competency Scale including subdimensions: technical skills, attitude and behaviors, estimation capacity, and foreign language skills	The study developed the Scientific Research Competency Scale with 57 items in four subdimensions. The reliability coefficients for the scale were high (Cronbach alpha: 0.98, Guttmann split-half: 0.96, and Spearman-Brown: 0.96). The breakpoint of the scale was determined to be 190, with a sensitivity of 72.76% and specificity of 67.16%.	Convenience sampling weakens the generalizability of the findings. A parallel form was absent in the language in which the scale was developed. Scale length may be a limitation in studies where the scale is applied in one-time measurement. The study ultimately included nursing students from only four universities, limiting the sample diversity. The absence of a parallel form may affect the validity and reliability of the scale. Scale length may pose challenges in studies with limited time for assessment. Convenience sampling may introduce bias and limit the representativeness of the findings.
Grande et al. [3], Saudi Arabia	Descriptive cross-sectional design	Total: 347	Completion of the Research Competency Scale for Nursing students (RCS-N) using the competency-based education (CBE) approach	The main findings of the study include a significant association between higher marks (A+/A) and older age (20 and above) with increased competency in nursing research. Additionally, there was a lack of familiarity with certain steps of the nursing process among participants, and competency-based education (CBE) was highlighted as beneficial for establishing learning outcomes effectively.	Research competencies of nursing students, academic performance (grades), and age	Participants with A+/A marks were three times more likely to be competent in nursing research compared to those with lower marks (OR = 2.81; 95% CI: 1.05-7.54; p = 0.04). Participants aged 20 and above had twice the probability of being research-competent compared to those under 20 (OR = 2.35; 95% CI: 1.30-4.24; p = 0.005).	Limited generalizability due to the involvement of only three universities. Cross-sectional design restricts establishing causality or relationships over time. Lack of additional research instruments like qualitative interviews for validation. Suggestions for further research include using quasi-experimental approaches or randomization of samples.
Tolentino et al. [14], Peru	Non-experimental, descriptive, correlational design; basic type; cross-sectional; substantive	Total: 236	The methodology used in the study included a hypothetical-deductive method, a quantitative approach, non-experimental, descriptive, correlational design, validated Likert-type questionnaires, and reliability checks through Cronbach's alpha values. Interventions are related to motivational factors, development of research skills, and cooperative learning methods.	There is a high level of motivational factors and investigative skills among nursing interns, with a significant positive correlation between the two. Internal factors such as family and teacher influence motivation and academic research performance. The relationship between motivational factors and investigative skills is crucial for the development of professional skills.	Levels of motivational factors and investigative skills among nursing interns	Not applicable	Not applicable
Qiu et al. [15], China	The study design is a psychometric study with repeated measurements.	Total: 146	The interventions that the study participants received were the Research Competency Scale for Nursing (RCS-N) at the beginning and the end of the nursing research course, a research methodology class, and they signed an informed consent form. The participants were from the top five nursing schools in China.	The RCS-N is a promising tool for evaluating research competency in undergraduate nursing students, but it cannot replace the actual examination of knowledge acquired in research methodology courses. The scale showed sensitivity to change after students participated in a research methods class, indicating an increase in research competency.	Research Competency Scale for Nursing students (RCS-N) scores before and after participation in the research methodology class	Before the nursing research class, the average score was 29.3, and after the class, it increased to 62.6, showing a significant improvement (p < 0.001). The effect size of the intervention was large (eta 2 = 0.82). There was no significant correlation between the RCS-N transformed total score and the class test score initially (r = 0.16, p = 0.051), but after excluding overly confident students, the correlation increased significantly to r = 0.45 (p < 0.001).	The limitations of the study include the small sample size, the focus on a specific nursing school limiting generalizability, the lack of testing for differential item functioning due to the small and homogenous sample, and the need to establish test-retest reliability for the RCS-N.

**Figure 1:**
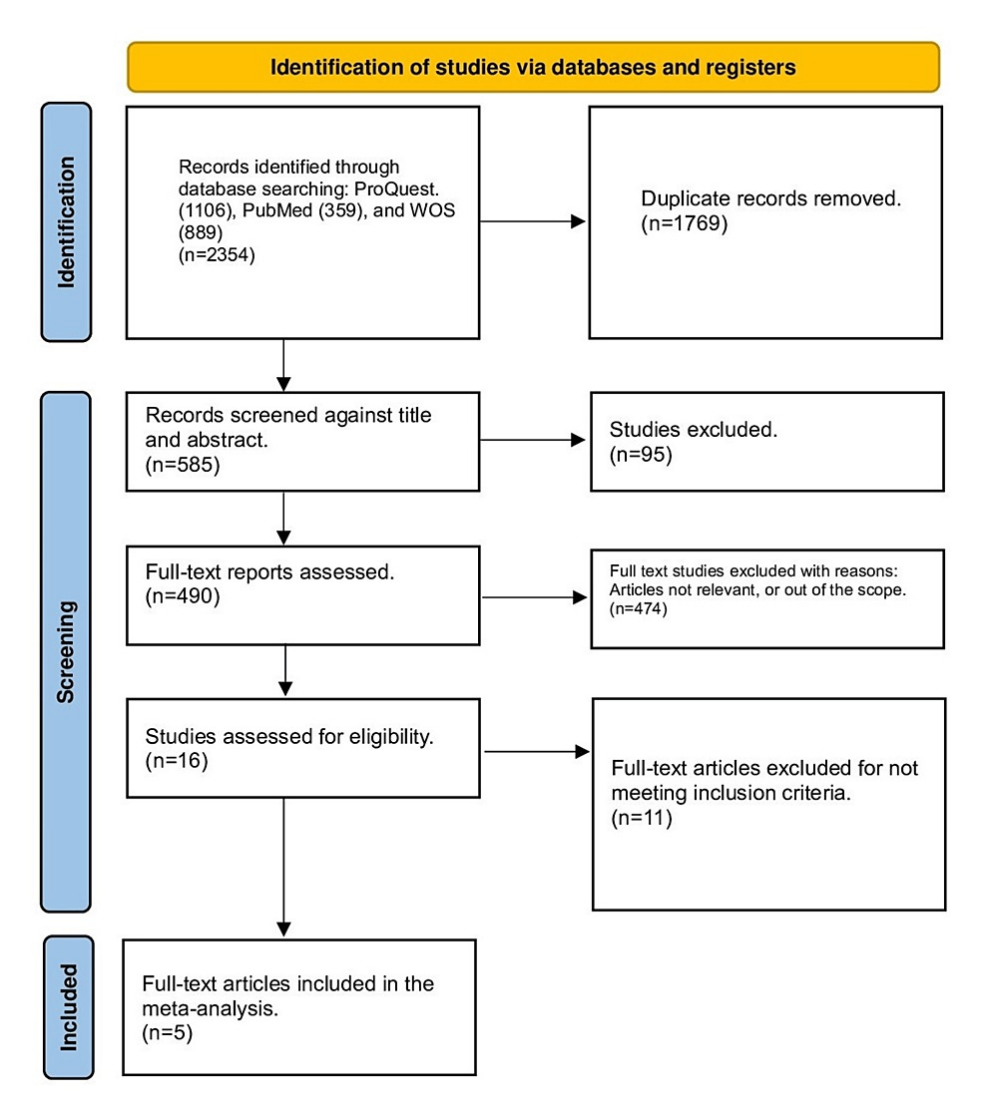
The PRISMA flow diagram PRISMA: Preferred Reporting Items for Systematic Reviews and Meta-Analyses.

The PRISMA flow chart shows the process of searching, screening, and selecting the studies (Figure 1). A total of 585 titles and abstracts were imported into an MS Excel spreadsheet for screening from the databases. After 95 duplicates were removed using Mendeley software, 490 full-text reports were reviewed for relevance. Of these, 474 studies were excluded as the articles were not relevant or out of scope. Sixteen studies were assessed for eligibility, and 11 full-text articles were excluded for not meeting inclusion criteria. In total, five studies were included in the final meta-analysis (Figure 1).

Quality Appraisal/Evaluation

The JBI checklist was used to rate the study's quality and determine the degree of bias in each study. To minimize errors and evaluate the methodological quality of each article, two reviewers examined all of the included studies in this systematic review. Every article had a computed score for participant selection, research methodology, and outcome evaluation, which was used to decide whether the study should be included in the final sample. Furthermore, the research quality of the quantitative designs was evaluated using the JBI critical appraisal tools [11]. Eight standards were used to assess the quality of the cross-sectional study. These standards are outlined as a checklist and are unique to quantitative data. Each study's quality ratings are considered based on the answers to a predefined set of questions (Table 3). Reviews indicated responses as "yes," "no," "unclear," or "not applicable" to these questions [11].

**Table 3:** Quality of the included studies

Item\Study	Duru et al. [13]	Grande et al. [3]	Qiu et al. [15]	Tolentino et al. [14]	Toraman et al. [12]
Were the criteria for inclusion in the sample clearly defined?	Yes	Yes	Yes	No	No
Were the study subjects and the setting described in detail?	Yes	Yes	Yes	No	No
Was the exposure measured in a valid and reliable way?	Yes	Yes	Yes	Yes	Na
Were objective, standard criteria used for measurement of the condition?	Yes	Yes	Yes	Yes	No
Were confounding factors identified?	No	Yes	No	No	No
Were strategies to deal with confounding factors stated?	No	Yes	No	No	No
Were the outcomes measured in a valid and reliable way?	Yes	Yes	Yes	Yes	Yes
Was appropriate statistical analysis used?	Yes	Yes	Yes	Yes	Yes

The studies that were included in the quality assessment revealed acceptable quality ranges (Table 3). Significant biases were discovered, nevertheless, as some studies failed to describe confounding variables and the methods used for controlling them.

Results

Search Outcomes

In March 2024, a search was conducted, in which five studies were selected after applying the inclusion and exclusion criteria and critically evaluating the studies. The sample population comprised 2191 nurses. The selected studies were published after 2016, with one in 2017, one in 2019, two in 2021, and one in 2023. Furthermore, of the five studies that were included, two were carried out in Turkey [12,13], one in Peru [14], one in Saudi Arabia [3], and one in China [15].

The Turkish study concluded that when comparing nursing students who did not take the course, those who did showed a greater degree of participation in research activities [12]. The percentage of students who attended scientific meetings, engaged in research activities, participated in research as subjects, and gathered data on behalf of a researcher was noticeably higher among those who took the research course. Nonetheless, the students in both groups demonstrated comparable levels of knowledge about and attitudes toward research [12].

A study conducted in China described the Research Competency Scale for Nursing (RCS-N), which was given to study participants at the beginning and end of the nursing research course [15]. The participants also attended a research methodology class and signed an informed consent form. One of China's top five nursing schools produced the participants. Before the research methodology class, the participants' RCS-N scores were 29.3, but after the class, their scores significantly improved to 62.6. However, the scale cannot replace the assessment of students' knowledge gained in research methodology courses, as evidenced by the lack of correlation found between the RCS-N result and class test scores [15].

Turkey was the location of the study's intervention [13]. The study developed the Scientific Research Competency Scale with 57 items in four subdimensions: technical skills, attitude and behaviors, estimation capacity, and foreign language skills. The reliability coefficients for the scale were high (Cronbach alpha: 0.98, Guttmann split-half: 0.96, and Spearman-Brown: 0.96). The breakpoint of the scale was determined to be 190, with a sensitivity of 72.76% and specificity of 67.16% [13].

The main finding of the research study conducted in Saudi Arabia was that the age and grades received in the nursing research course were strongly correlated with the research competencies of nursing students [3]. Furthermore, the likelihood of research competency was twice as high for nursing students over the age of 20 as for those under 20. These results underscore the necessity of assessing relevant learning outcomes and the significance of incorporating CBE approaches into nursing research education [3].

The most important finding of the Peruvian research study is that among nursing interns enrolled in a university degree program, there is a highly significant positive correlation between investigative skills and motivational factors [14]. Most of the participants demonstrated strong levels of both their capacity for investigation and motivation. Using Spearman's Rho, the correlation between these variables was ascertained. A strong positive relationship was indicated by a Rho value of 0.944 and a p-value of 0.003 [14].

Meta-analysis results

Data Transformation

Correlation coefficients were taken from each study and were transformed using Fisher’s Z-transformation. This was followed by the computation of the effect size and standard error. For studies that reported frequency tables, data was transformed to Phi correlation and then to effect size transformation. The main outcome of the study was to assess the attitude, behavior, test score, and motivational factors toward research skills and competency among nursing students (Table 4).

**Table 4:** Data transformation table r: Correlation coefficients; S.E.: Standard error; ES: Effect size.

Study ID	Sample size	r	Variance r	S.E.	ES	Variance of ES	S.E. of ES	Outcome type
Tolentino et al. [14]	236	0.926	8.64387E-05	0.009297	1.629568	0.004115226	0.06415	Motivational toward research
Duru et al. [13]	937	0.897	4.07881E-05	0.006387	1.45665	0.001070664	0.032721	Attitude and behavior toward research
Qiu et al. [15]	149	0.16	0.006415239	0.080095	0.161387	0.006849315	0.082761	Preform class test results in research
Grande et al. [3]	347	0.162	0.002740465	0.052349	0.16344	0.002906977	0.053916	Preform test score results in research
Toraman et al. [12]	522	0.008	0.00191914	0.043808	0.015	0.001926782	0.043895	Preformed research

Analysis Process

Data were analyzed using SPSS v28 software (IBM Corp., Armonk, NY). Effect size and standard error were reported for those who took research courses or training. Cochran’s Q test and I^2^ statistics were used to assess heterogeneity between studies. If the P-value of Cochran’s Q test is significant and I^2^ statistics are higher than 50%, the random-effect model will be applied. Otherwise, a fixed effect model will be suitable. The outcome will be a forest plot and funnel plot for overall effect size. Publication bias was assessed using Egger’s test.

Analytical Findings

**Figure 2:**
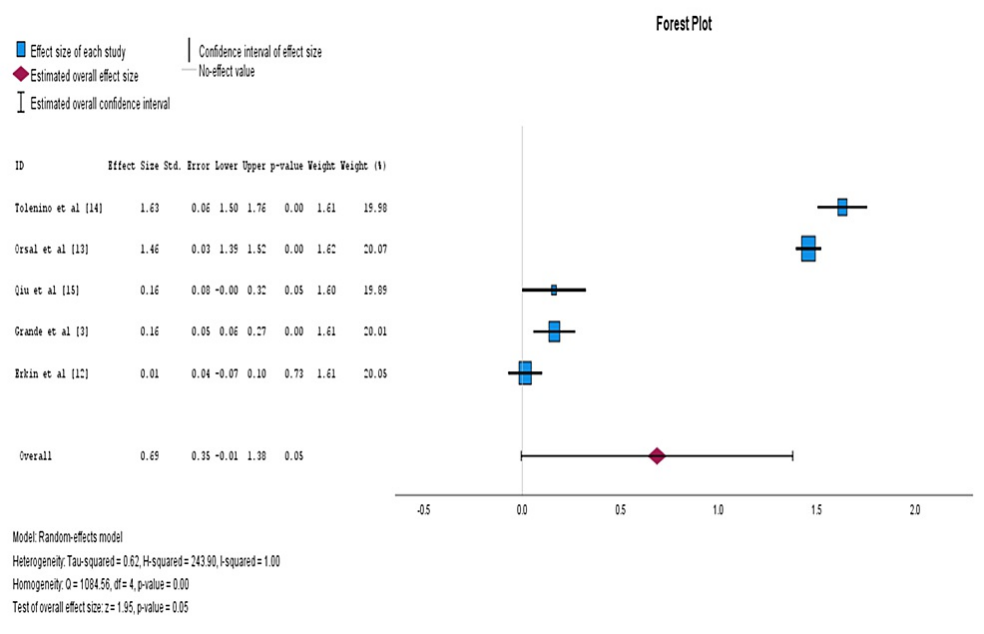
Forest plot for meta-analysis with fixed effects model

As I^2^ was higher than 50% and Cochran’s Q test was significant, a random-effect model was used. Egger’s test showed that publication bias was not present in this meta-analysis. The total number of studies included was five. The effect size was 0.69 ± 0.35 (P = 0.05), which indicates a high effect on research competency among nursing students who attend courses or training in research matters, after transforming data to correlation coefficient resulting in r = 0.5 (Figure 2).

Discussion

The meta-analysis synthesized data from five studies to evaluate the impact of CBL on the assessment of nursing students' research skills. The findings suggest that CBL positively affects students' engagement with research activities, improves their competencies in specific areas of nursing research, and enhances their motivation and attitude toward research.

Studies included in this meta-analysis [12,3] provide empirical support for the argument that CBL not only enhances specific research skills but also promotes greater engagement in research activities. For instance, students enrolled in a research course under a CBL framework participated more actively in scientific meetings and research tasks compared to their counterparts who did not take the course. This suggests that CBL effectively bridges the gap between theoretical learning and practical application, a critical aspect of nursing education that traditional models often overlook [12].

This meta-analysis highlights the uniformity in the positive impact of CBL across diverse studies, indicating its robustness in enhancing research skills among nursing students. However, when these findings are compared with real literature studies, interesting nuances emerge, which contextualize these results within a broader educational framework. For example, the study by Grande et al. [3] reported that older students and those with higher academic achievements displayed better research competencies. This aligns with findings from a study by Lewis et al. [5], which observed that maturity and prior academic exposure to research concepts could enhance the effectiveness of CBL. This suggests that while CBL is effective, its impact can be influenced by the demographic and academic backgrounds of the learners, which can lead to variations in outcomes across different student populations.

Additionally, the positive correlation between motivational factors and investigative skills noted by Tolentino et al. [14] is echoed in the literature by [14], who found that motivation plays a critical role in the acquisition of competencies. Both studies underscore the importance of internal and external motivational factors in fostering a conducive learning environment for CBL. This is indicative of the broader educational principle that student engagement and motivation are critical drivers of success in competency-based frameworks.

Furthermore, the Turkish studies included in the meta-analysis [12,13] contribute to an understanding of how cultural and institutional contexts can affect the implementation and outcomes of CBL. This is comparable to the findings by Vasquez et al. [8], which suggested that the effectiveness of CBL could vary significantly depending on the institutional support and cultural attitudes toward independent learning and competency assessment.

The meta-analysis results demonstrate some notable differences when compared with findings from other educational research studies, particularly in how CBL impacts various aspects of nursing education. Additionally, the findings from Qiu et al. [15] that CBL does not completely replace the need for traditional assessments in evaluating complex research skills highlight a unique challenge in nursing education. In other disciplines, such as engineering or business, CBL has been shown to successfully replace more traditional forms of assessment, with competency demonstrations providing a holistic measure of student ability [9]. This difference could be attributed to the nature of nursing as a field that combines technical skills with critical human-centered care, requiring both competency achievement and traditional knowledge assessments to ensure comprehensive training.

These differences underscore the necessity of a nuanced approach to implementing CBL in nursing education. While CBL can enhance certain educational outcomes, it must be integrated thoughtfully alongside traditional methodologies to fully address the specific needs of nursing students.

Implication of Nursing Research Skills

Through the assessment of research skills, nursing education can guarantee that students possess the requisite knowledge and skills to evaluate and apply research findings critically in their clinical practice. Furthermore, focusing on research skills in nursing education can result in better patient outcomes. While making evidence-based decisions, nurses with research competencies can actively incorporate the most recent research findings into their clinical practice. Finally, nurses with strong research abilities can influence health policy and practice. Evidence-based guidelines, protocols, and standards of care can benefit from their critical evaluation and application of research findings. Policy decisions and advancements in nursing practice can be influenced by the insightful information that nursing research can offer on patient safety, healthcare delivery, and health promotion.

Limitations of the study

The first limitation is the insufficient number of included studies. Furthermore, even though this systematic review used multiple MeSH terms and three databases, it is possible that other studies from other databases were left out. Lastly, as each included study had a cross-sectional design, there were fewer options for analysis to indicate how effectively competency-based learning improved nursing students' assessment of their research skills.

## Conclusions

The study encourages research literacy among nursing students. Through competency-based learning, students are exposed to a variety of research methodologies, ethical issues, and scientific writing conventions. This exposure enhances their capacity to understand, assess, and apply research evidence, empowering them to become knowledgeable consumers and field contributors.
